# Microparticles-Mediated Vascular Inflammation and its Amelioration by Antioxidant Activity of Baicalin

**DOI:** 10.3390/antiox9090890

**Published:** 2020-09-20

**Authors:** Keshav Raj Paudel, Dong-Wook Kim

**Affiliations:** Department of Oriental Medicine Resources, Mokpo National University, Muan-gun, Jeonnam 534-729, Korea; Keshavrajpaudel@gmail.com

**Keywords:** microparticles, baicalin, atherosclerosis, migration, proliferation, inflammation

## Abstract

Microparticles (MPs) are extracellular vesicles (0.1–1.0 μm in size), released in response to cell activation or apoptosis. Endothelial microparticles (EC-MP), vascular smooth muscle cell microparticles (VSMC-MP), and macrophage microparticles (MØ-MP) are key hallmarks of atherosclerosis progression. In our current study, we investigated the potent antioxidant activity of baicalin to ameliorate MP-induced vascular smooth muscle cell (VSMC) dysfunction and endothelial cell (EC) dysfunction, as well as the production of inflammatory mediators in macrophage (RAW264.7). In our study, baicalin suppressed the apoptosis, reactive oxygen species (ROS) generation, NO production, foam cell formation, protein expression of inducible nitric oxide synthase and cyclooxygenase-2 in MØ-MP-induced RAW264.7. In addition, VSMC migration induced by VSMC-MP was dose-dependently inhibited by baicalin. Likewise, baicalin inhibits metalloproteinase-9 expression and suppresses VSMC-MP-induced VSMC proliferation by down-regulation of mitogen-activated protein kinase and proliferating cell nuclear antigen protein expressions. Baicalin also inhibited ROS production and apoptosis in VSMC. In EC, the marker of endothelial dysfunction (endothelial senescence, upregulation of ICAM, and ROS production) induced by EC-MP was halted by baicalin. Our results suggested that baicalin exerts potent biological activity to restore the function of EC and VSMC altered by their corresponding microparticles and inhibits the release of inflammation markers from activated macrophages.

## 1. Introduction

Microparticles (MPs) are extracellular vesicles with a size ranging from 0.1 to 1.0 μm. They carry cargo (mRNA, DNA, lipid and specific proteins) from originating cells and transfer to recipient cells, allowing cell-to-cell communication. MP release is triggered by inducer that can cause cell apoptosis or activation [1] and are generated by any type of body cells such as platelets, endothelial cells, leukocytes, smooth muscle cells, and erythrocytes [2]. In the past, MPs were considered as “cell debris/dust” but now, it is clear that MPs are not just cell debris; however, the detailed process of their generation is yet to be elucidated [3]. Nowadays, it is known that the release of MPs from cells involves the reconstitution of a phospholipid bilayer with the outside of the membrane exposed with phosphatidylserine and change in the cellular organization with the disruption of cytoskeleton architecture [4]. Recently, the role of MPs in the pathogenesis of central nervous system disorder [5], diabetes mellitus [6], cancer [7], inflammation [8], systemic lupus erythematosus [9], endothelial dysfunctions [10] has been explored by clinical/experimental research. In the human systemic circulation, elevated levels of endothelial MPs (EC-MP) are linked with the progression of various cardiovascular diseases, primarily initiated by endothelial dysfunction [11]. An in vitro study revealed that EC-MP treatment affects the various angiogenesis parameters by reducing endothelial cell (EC) proliferation, decreasing capillary formation and increasing apoptosis [12]. Hidenobu Koga et al. observed a higher level of the cluster of differentiation (CD)-144 or vascular endothelial (VE)-cadherin positive EC-MP in the systemic circulation of patients with diabetic mellitus and coronary artery disease, including atherosclerosis. This suggests VE-cadherin positive EC-MP in serum can be a hallmark for analyzing atherosclerosis. Thus, various cardiovascular complications can be efficiently prevented by the therapeutics approach in atherosclerosis patients focusing on assessments of blood CD144-EMP levels [13].

Microparticles are produced by macrophage cells in response to various stimuli such as lipopolysaccharide (LPS), poly (I:C), actinomycin D [14,15]. LPS is a toll-like receptor (TLR)-4 ligand, and poly (I:C) is a TLR3 ligand that can stimulate MØ-MP via TLR. MØ-MP release induced by LPS or poly(I:C) is correlated with nitric oxide (NO) production, and treatment with the inducible nitric oxide synthase (iNOS) inhibitor 1400W decreased particle release and NO production. Furthermore, the treatment of RAW 264.7 cells with NO donors induced MPs production [14]. MPs from activated macrophages could carry tumor necrosis factor-alpha (TNF-α) and contribute to the propagation of inflammatory signals leading to myocardial infarction. Milbank E et al. hypothesized that MPs from human carotid atherosclerotic plaques might contain active TNF-α, which could contribute to MPs-induced inflammatory signals in human atherosclerotic lesions [15].

Pro-atherogenic inducer such as TNF-α, thrombin, and lysophosphatidylcholine can generate MPs by vascular smooth muscle cells (VSMC) [16]. VSMC-microparticles (VSMC-MP) possesses protein such as caveolin-1 [16] and alpha-smooth muscle actin (α-SMA) [17] obtained from originating VSMC, making it delectable in an experimental setting by labeling VSMC-MP with either caveolin-1 or α-SMA. EC, VSMC and macrophage are three important cell types involved in the pathogenesis of atherosclerosis either in initiation steps or later progression of vascular inflammation [16]. G. Chiva-Blanch et al. found that aspirin therapy inhibits vascular wall cell activation and microparticle shedding by VSMC, suggesting a therapeutic target to lower microparticles released from cells can prevent the progression of the disease such as diabetes mellitus and atherosclerosis [17]. Additionally, MPs generated from apoptotic VSMC (commonly observed in atherosclerotic plaques) can, in turn, induce endothelial dysfunction as shown by its decreased nitric oxide (NO) production and vasodilatory capacity [18]. Previously, we have shown that VSMC-MP promotes the proliferation of VSMC through the upregulation of the mitogen-activated protein kinase pathway (MAPK) and proliferative cell nuclear antigen (PCNA) to facilitate vascular inflammation [19]. Similarly, in a recent review paper, we have highlighted the role of circulating EC-MP in the progression of atherosclerosis through oxidative stress, and upregulation of iNOS, cyclooxygenase (COX-2) and nuclear factor kappaB (NF-κB) pathway [20]. As cell-to-cell communication mediated by cargos of microparticles has been recently explored, there exists a potential research platform to investigate potent drug candidates that can target various pathways to alleviate the disease progression. In this aspect, plant-based phytochemicals are one of the emerging therapeutic alternatives.

Baicalin is a flavonoid found in *Scutellaria baicalensis* Georgi. It is a well-known antioxidant and anti-inflammatory compound showing its therapeutic potential in cardiovascular diseases, diabetes, asthma, hyperuricemia, rheumatoid arthritis, and cancer. The antioxidant activity of baicalin is due to its potency to neutralize ROS, whereas its anti-inflammatory capacity is because of the inhibition of the NF-κB pathway thereby attenuating the expression of several inflammatory mediators as well as decreased expression of iNOS, COX-2, lipoxygenases, cell adhesion molecules, TNF-α, and interleukins [21,22]. Since endothelial dysfunction, the release of inflammatory mediators from macrophages and proliferation and migration of VSMC are key events leading to vascular inflammation [23], we sought to target these main events of atherosclerosis progression by inducing cells with microparticles. Therefore, the objective of this study was to evaluate the antioxidant activity of baicalin to ameliorate vascular smooth muscle cell and endothelial dysfunction.

## 2. Materials and Methods

### 2.1. Cell-Free In Vitro Experiments

#### 2.1.1. Chemicals and Reagents

Baicalin was purchased from Sigma Aldrich, USA. Similarly, DPPH, ferrous sulphate, 2,2′-bipyridyl, copper sulphate, thiobarbituric acid, 1,1,3,3,-tetraethoxypropane, methanol, and butanol were purchased from Sigma, USA. All the other chemicals and reagents were of reagent grade and purchased from Sigma Aldrich, USA, unless stated.

#### 2.1.2. Antioxidant Assay

DPPH radical scavenging activity, chelation assay, and TBARS assay were carried out as described previously [24].

### 2.2. Cell-Based In Vitro Experiments

#### 2.2.1. Cell Culture and Reagents

Endothelial cells were isolated from the porcine coronary artery as described previously [25]. Vascular smooth muscle cells (VSMC) were purchased from the American Type Culture Collection (ATCC, Manassas, VA, USA), and RAW264.7 (immortalized murine macrophages cell line obtained from BALB/c mice with Abelson murine leukemia virus-induced tumor) was purchased from the Korean cell line bank (Seoul, Republic of Korea) and grown at 37°C under a humidified, 5% CO_2_ atmosphere in Dulbecco’s modified eagle’s media (Lonza, Walkersville, MD USA) supplemented with 10%FBS, 2mM glutamine, 100 units/mL of penicillin, 100 µg/mL of streptomycin and 2.5 µg/mL of amphotericin B. p44/42 (ERK1/2), phospho p44/42 (pERK1/2), SAPK/JNK, phospho SAPK/JNK, p38, phospho p38, MMP-9, phospho ENOS, ENOS, phospho Akt, Akt, iNOS, COX-2 and β-actin was purchased from Cell Signalling Technology (Danvers, MA, USA). PCNA primary antibody was purchase from Abcam (Cambridge, MA, USA). Intercellular adhesion molecule 1 (CD54/ICAM-1), Alexa488 and Alexa555 were purchase from Biocompare (San Francisco, CA, USA). Annexin V-FITC Apoptosis Detection Kit was purchased from BioBud (Seoul, Republic of Korea), 4’,6-diamidino-2-phenylindole (DAPI) was purchased from Thermo Fisher Scientific (Waltham, MA, USA). 5-dodecanoylaminofluorescein di-β-d-galactopyranoside was purchased from Invitrogen (Illkirch, France). 3-(4, 5-dimethylthiazol-2-yl)-2, 5 diphenyltetrazolium bromide (MTT), 2′,7′-dihydrodichlorofluorescin diacetate (DCF-DA), Oil Red O staining solution, DMSO, phalloidin-FITC were purchased from Sigma-Aldrich (St Louis, MO, USA).

#### 2.2.2. Isolation, Characterization, and Quantification of Microparticles.

Isolation of MPs was performed according to the protocol described by Boulanger et al., 2001 [26]. As microparticles are released by the process of cell activation or apoptosis, researchers use various stimulants in high concentrations for high-yield production of microparticles. Gauley et al. treated LPS at a dose ranging from 0.05 to 50 µg/mL in the RAW264.7 cell line and found the highest yield of microparticles and a corresponding increase in nitrite (a marker of inflammation) at 50 µg/mL LPS [14]. In our study, we also use high doses of LPS for EC and RAW264.7 for the high yield of microparticles. Briefly, confluent EC and RAW264.7 were stimulated with 10 µg/mL and 50 µg/mL LPS respectively for 24 h. Confluent VSMC were stimulated with 20 ng/mL of TNF-α for 16 h [16]. The cell supernatant from these stimulated cells (Five 100mm cell culture plates for each cell) was collected and subjected to multiple centrifugation steps to obtain an MP pellet of respective cells and the process was repeated to obtain enough MPs for all experiments. Each cell MP was pooled into a single Eppendorf tube mixed in PBS and stored at -70°C until further use.

Characterization of microparticles was carried out by immunofluorescence. EC-MP was stained with ICAM primary antibody followed by Alexa fluor-488 secondary antibody [27], VSMC-MP were stained with α-SMA, followed by Alexa fluor-488 secondary antibody [17], and MØ-MP were stained with annexin V-FITC as a general marker of microparticles [14]. Fluorescence images were taken at different magnifications using confocal or microscope.

Quantification of microparticles was performed by measuring protein concentration. For each cell microparticle, 10 µL of MP was mixed with 200 µL of 20% Bio-Rad protein assay reagent, and absorbance of resultant blue color was read at 570nm. The protein concentration of microparticles in terms of mg/mL was calculated with reference to the standard curve equation obtained from a serial dilution of bovine serum albumin. For subsequent in vitro cell experiment, respective cell MPs at a concentration of 10 µg/mL protein equivalent were used to induce the cell.

#### 2.2.3. Cell Viability Assay

The viability of RAW 264.7 cells, VSMC and EC treated with respective MPs, and a various dose of baicalin was determined by assaying the reduction of 3-(4, 5-dimethylthiazol-2-yl)-2, 5-diphenyltetrazolium bromide (MTT) to formazan by viable cells as described previously [24].

#### 2.2.4. NO Production

NO production in MØ-MP-stimulated RAW 264.7 cell was determined by the Griess reagent method as described previously [24].

#### 2.2.5. Foam Cell Assay

Foam cell assay was carried out in RAW264.7 cell as described previously with slight modification [24]. Oil Red O (ORO) staining was performed in oxidized-low density lipoprotein (ox-LDL) and/or baicalin with/without 10 μg/mL of MØ-MP treated RAW264.7 cell. Positive stained (red) foam cells were observed via phase-contrast microscope (TE200; Nikon, Tokyo, Japan). Photographs were taken at 40 × magnification. Colorimetric quantification of lipid content in oxidized LDL and/or MØ-MP treated RAW 264.7 cells after ORO staining was performed.

#### 2.2.6. Western Blot

RAW264.7 cell, EC, and VSMC were treated with or without 10 μg/mL protein equivalent of MØ-MP (for RAW264.7), 10 μg/mL protein equivalent of EC-MP (for EC), 10 μg/mL protein equivalent of VSMC-MP (for VSMC), and various doses of baicalin. Cell lysate (30 μg/mL protein) was subjected to SDS-PAGE for detection of the following protein; iNOS and COX-2 protein expression were detected in RAW 264.7 cell, MAPK signaling protein, MMP-9, PCNA protein expression were detected in VSMC and ICAM, ENOS, Akt were detected in EC by the Western blot method as described previously [24].

#### 2.2.7. ROS Formation

**a. Measurement of ROS by fluorescence intensity quantification:** RAW264.7 cells, EC, VSMC were cultured in black 96-well plate. Cells were treated with or without a various dose of baicalin and 10 μg/mL protein equivalent of MØ-MP (for RAW264.7), 10 μg/mL protein equivalent of EC-MP (for EC), and 10 μg/mL protein equivalent of VSMC-MP (for VSMC) for 4 h. An amount of 10 μM of 2′,7′-dihydrodichlorofluorescin diacetate was added for 60 min in the dark. Cells were washed with PBS, and fluorescence intensity was measured at excitation 485 nm and emission 527 nm [28,29,30].

**b. Measurement of ROS by fluorescence imaging:** RAW264.7 cell, EC, VSMC was cultured in a Cell Tek solution-coated glass slide. Cells were treated with or without a various dose of baicalin and 10 μg/mL protein equivalent of MØ-MP (for RAW264.7), 10 μg/mL protein equivalent of EC-MP (for EC), and 10 μg/mL protein equivalent of VSMC-MP (for VSMC) for 4 h. An amount of 10 μM of 2′,7′-dihydrodichlorofluorescin diacetate was added for 5 min in the dark. Cells were washed with PBS, and images were taken immediately with a fluorescence microscope [28,29,30].

#### 2.2.8. Apoptosis Assay

Baicalin was treated with RAW264.7 cells, EC and VSMC at a different concentration for 1 h followed by stimulation with 10 μg/mL protein equivalent of MØ-MP (for RAW264.7), 10 μg/mL protein equivalent of EC-MP (for EC), and 10 μg/mL protein equivalent of VSMC-MP (for VSMC) for 24 h. Cells were centrifuged at 1500 rpm for 4 min. Supernatants were discarded was cell pellets were washed with PBS 1 time followed by incubation of cell in Annexin V-FITC solution, prepared in 1X binding buffer, for 30 min in the dark. After centrifugation at 1000 g for 5 min, cells were incubated in propidium iodide prepared in 1X binding buffer for 15 min at 4°C. Analysis of apoptosis was performed with Arthur^TM^ image-based cell analyzer (NanoEnTek, Seoul, South Korea) [31,32].

#### 2.2.9. VSMC Proliferation Assay

The proliferation of VSMC, induced by VSMC-MP in the presence of various doses of baicalin, was determined by assaying the reduction of 3-(4, 5-dimethylthiazol-2-yl)-2, 5-diphenyltetrazolium bromide (MTT) to formazan by viable cells as described previously [24]. The proliferation rate of the control group (only VSMC-MP treated) was considered 100%, while the effect of the baicalin on proliferation was evaluated as % of control.

#### 2.2.10. Immunocytochemistry (PCNA Expression in VSMC and β-Galactosidase Staining in EC)

Baicalin was treated with VSMC at a different concentration for 1 h, followed by stimulation with 10 μg/mL of VSMC-MP for 24 h. Cells were washed three times with PBS and blocked with 5% BSA followed by incubation of PCNA primary antibody in the dark overnight. After washing with PBS, cells were incubated with Alexa 555 and DAPI secondary antibody for 1 h. Fluorescence images were taken with a fluorescence microscope.

EC at passage one was treated with 10 μM of baicalin and/or 10 μg/mL of EC-MP for 24 h. After pretreatment with chloroquine for 1 h, cells were labeled with 5-dodecanoylaminofluorescein di-β-d-galactopyranoside for 1 h, washed with cold PBS, fixed with 4% buffered formalin, permeabilized with 1% Triton X-100, blocked with 2.5% bovine serum albumin, followed by DAPI for nuclear stain. Fluorescence images were taken at 20X magnification with a confocal microscope [33,34].

#### 2.2.11. Gelatin Zymography

VSMC, seeded at the concentration of 5 × 10^5^ cells in a 60 mm dish, were starved in 0.4% FBS/DMEM for 24 h. Then, baicalin was treated at a different concentration for 1 h, followed by stimulation with 10 μg/mL of VSMC-MP for 24 h. The conditioned medium of cells was subjected to gelatin zymography to detect MMPs enzymatic activity as described previously [24,35].

#### 2.2.12. Boyden Chamber Assay

VSMC migration assay was performed using a modified Boyden chamber as described previously with slight modification. VSMC cells were seeded at a density of 1 × 10^5^cells/mL in 200 µL of DMEM containing 0.1% BSA in the upper chamber while the lower chamber has 600 µL of DMEM with or without 10 μg/mL of VSMC-MP along with a different concentration of baicalin. The cells were allowed to migrate for 48 h. The cells that had migrated to the lower face of the membrane were fixed in 10% formalin and stained with a hematoxylin and eosin dye. The cell migration images were taken with phase-contrast microscopy. Stained cells possessing a distinct nucleus were counted in five random fields of view (magnification x 20) and calculated as cells per field of view [24].

#### 2.2.13. Wound Healing Assay

VSMC, seeded at the concentration of 5x10^5^ cells /mL, were starved in 0.4% FBS/DMEM for 24 h. The monolayer was scratched with a yellow tip to create a migrating zone and then washed with PBS to remove debris. Photographs were taken at time 0. Then, 10 μg/mL VSMC-MP and baicalin at the concentrations of 10 and 25 µM was treated for 48 h. The migrating cells were photographed by a light microscope at the magnification of 20X [24].

### 2.3. Statistical Analysis

All data were expressed as mean ± SE. The significance of variance among different groups was determined by one-way analysis of variance (ANOVA) followed by Dunnett multiple comparison test. *p-*value ≤ 0.05 were considered to be significantly different.

## 3. Results

### 3.1. Characterization of Microparticles

**Figure 1:**
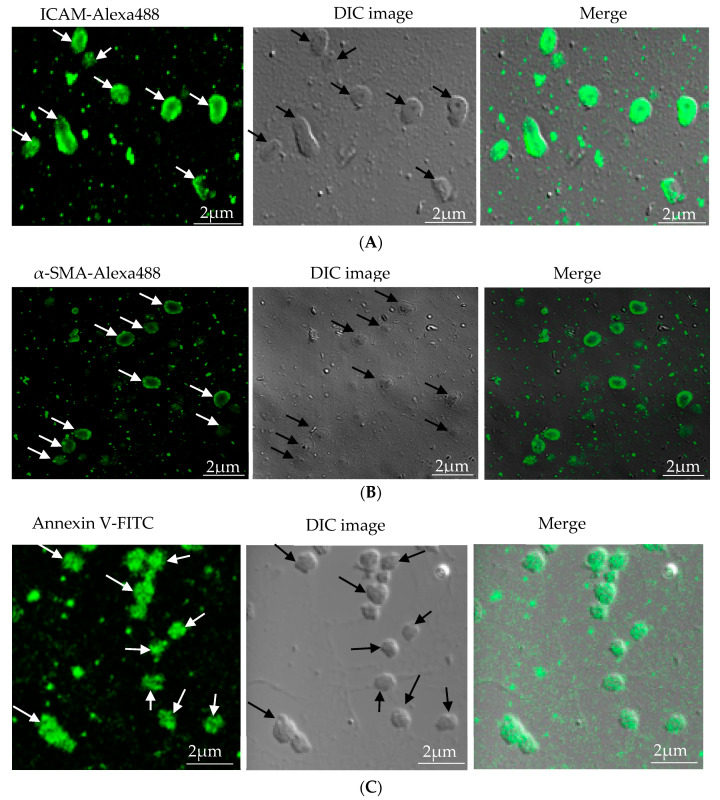
Characterization of microparticles isolated from various cell lines. (**A**) Endothelial microparticles (EC-MP) (shown by arrowhead) obtained from in vitro lipopolysaccharide (LPS)-treated endothelial cell (EC) were stained with ICAM followed by Alexa-488 and photographs were taken with a confocal microscope at a magnification of 630 X Zoom 4. (**B**) Vascular smooth muscle cell (VSMC)-MP (shown by arrowhead) obtained from tumor necrosis factor-alpha (TNF-α)-stimulated VSMC were stained with α-smooth muscle actin (α-SMA), and photographs were taken with a confocal microscope at a magnification of 630 X Zoom. (**C**) Macrophage microparticles (MØ-MP) obtained from LPS treated macrophage (RAW264.7) were stained with Annexin V-FITC, and photographs were taken with a confocal microscope at a magnification of 630 X Zoom 4.

Microparticles were successfully isolated from VSMC and macrophage and EC, in vitro. Figure 1A represents the confocal microscopic image of in vitro isolated EC-MP stained with ICAM-Alexa 488. Similarly, Figure 1B (copyright@American Scientific Publishers) represents the confocal image of VSMC-MP stained with α-SMA-Alexa 488 [19]. Likewise, Figure 1C represents the confocal image of Annexin-V-FITC labeled MØ-MP generated in vitro from macrophages (RAW264.7 cells).

### 3.2. Baicalin Inhibits NO Production, iNOS, and COX-2 Protein Expression in MØ-MP Induced RAW264.7 Cell

**Figure 2:**
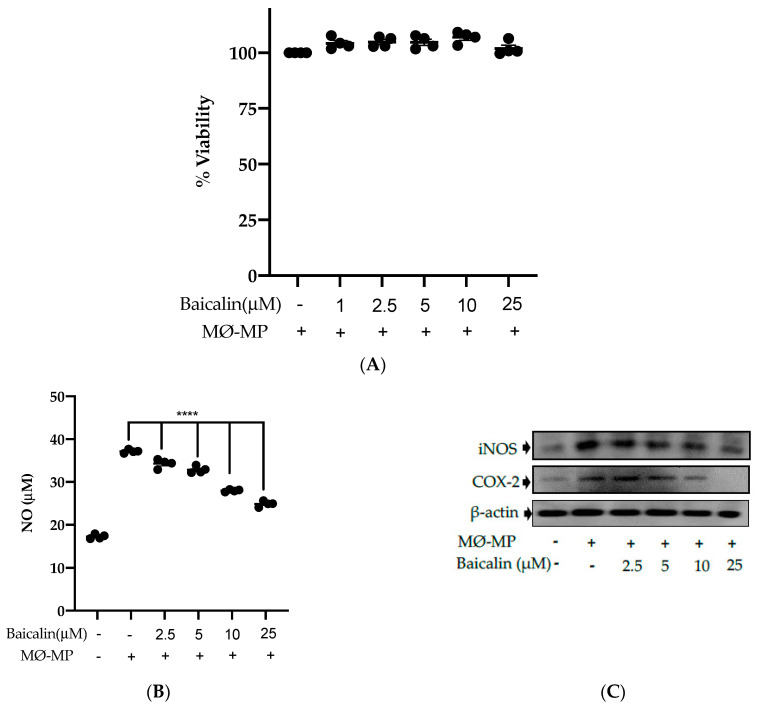

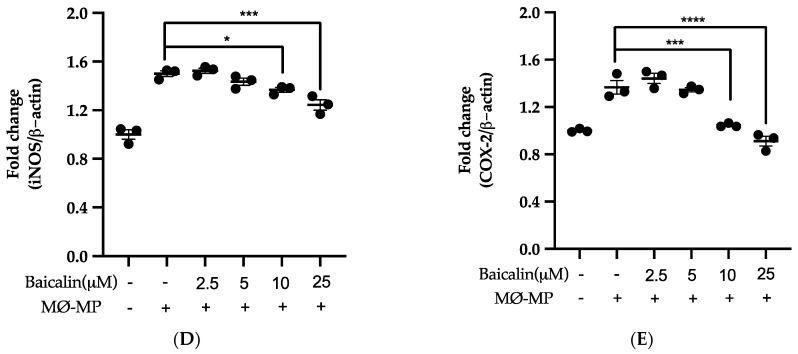
Effect of baicalin on nitric oxide (NO) production, iNOS, COX-2 protein expression in RAW 264.7 cell. (**A**) RAW264.7 cell was incubated with MØ-MP and a various dose of baicalin for 24 h, followed by MTT solution for 4 h. The absorbance of purple formazan was taken at 540 nm. (**B**) RAW264.7 cell was treated with or without MØ-MP and various doses of baicalin for 24 h. The NO release by activated RAW264.7 in the supernatant media was mixed with an equal volume of Griess reagent and absorbance of resultant color was taken at 540 nm. (**C**) The cell lysate of RAW264.7 treated with or without MØ-MP and various doses of baicalin for 24 h were subjected to SDS-PAGE for the detection of iNOS and COX-2 protein expression by Western blot. β-actin was used for normalization. (**D**) Quantitative data of iNOS fold change. (**E**) Quantitative data of COX-2-fold change. Each value is the mean ± SE of three to four independent experiments and each independent experiment contains five replicates. *p < 0.05, ***p < 0.001, ****p < 0.0001 vs. control (MØ-MP alone). The analysis was performed using one-way ANOVA followed by Dunnett multiple comparison test.

Firstly, we evaluated the RAW264.7 cell viability after treatment with various doses of baicalin and MØ-MP. As shown in Figure 2A, baicalin, at a dose of up to 25 µM, did not alter the RAW264.7 cell viability compared to normal (baicalin untreated group). Figure 2B shows the efficacy of baicalin to inhibit the NO production in MØ-MP induced RAW264.7 cells, whereas Figure 2C–E shows the potency of baicalin to reduce the protein expression of iNOS and COX-2.

### 3.3. Baicalin Inhibits MØ-MP Induced ROS Production, Apoptosis and Foam Cell Formation in RAW264.7 Cell

**Figure 3:**
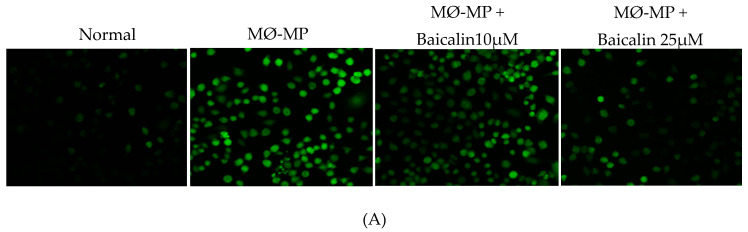

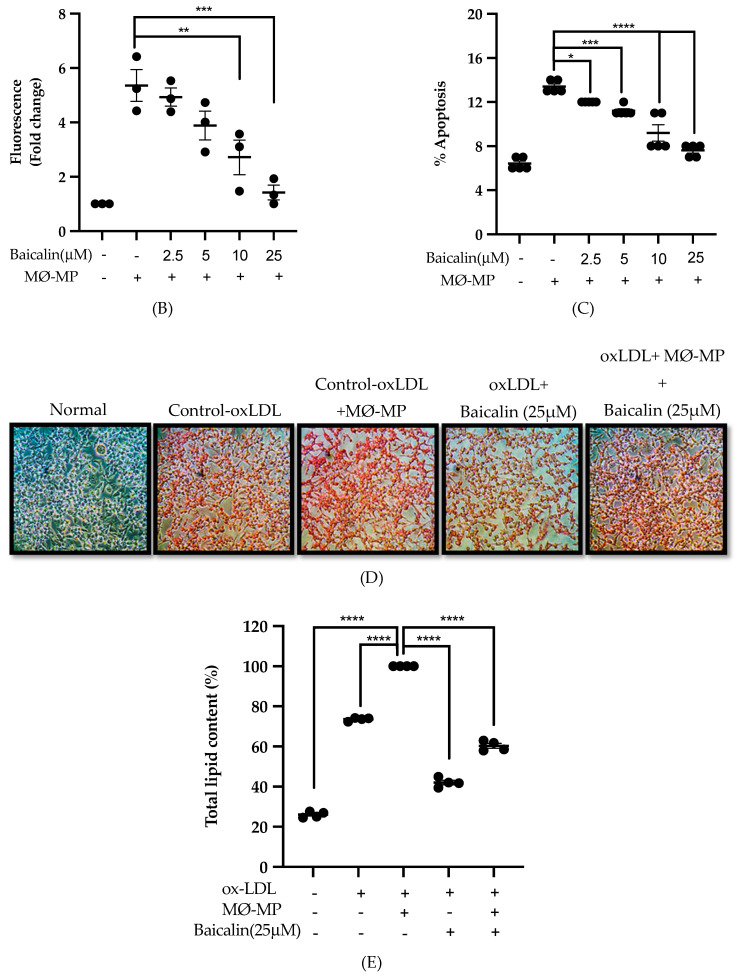
Effect of baicalin on MØ-MP induced reactive oxygen species (ROS) production, apoptosis, and foam cell formation in RAW264.7. (**A**) RAW264.7 cell was treated with or without MØ-MP and a various dose of baicalin for 24 h. DCF-DA was treated to detect the ROS produce by activated RAW264.7 indicated by green fluorescence. Image was taken at 20× magnification. (**B**) ROS fluorescence intensity was measured at excitation 485 nm and emission 527 nm. (**C**) RAW264.7 cell was treated with or without MØ-MP and a various dose of baicalin for 24 h. Apoptosis assay of RAW264.7 cell measured by Annexin V-FITC staining. (**D**) RAW264.7 cell was treated with or without ox-LDL, MØ-MP and baicalin. The ox-LDL engulfed by cells was measured by Oil Red O staining. (**E**) After ORO, the cell was rinsed with 60% isopropyl alcohol, and the lipid content was quantified. Each value is the mean ± SE of three to five independent experiments and each independent experiment contains five replicates. *p < 0.05, **p < 0.01, ***p < 0.001, ****p < 0.0001 vs. control (MØ-MP alone). The analysis was performed using one-way ANOVA followed by Dunnett multiple comparison test.

As shown in Figure 3A,B, baicalin dose-dependently inhibited the ROS production in MØ-MP induced RAW264.7 cell. In consistence with a decrease in ROS production, there was a dose-dependent inhibition of RAW264.7 cell apoptosis by baicalin (Figure 3C). Similarly, the oxidized-LDL intake by RAW264.7 is increased by treatment with MØ-MP revealed by increased intensity of Oil Red O-staining of the cell. However, baicalin, at a dose of 25 µM, decreased the ox-LDL intake (Figure 3D). Consistently, the lipid quantification after foam cell assay shows the high lipid content in RAW264.7 cells treated with ox-LDL and MØ-MP whereas baicalin decreased the lipid accumulation inside RAW264.7 (Figure 3E).

### 3.4. Baicalin Target the Endothelial Dysfunction Marker to Restore EC Function

**Figure 4:**
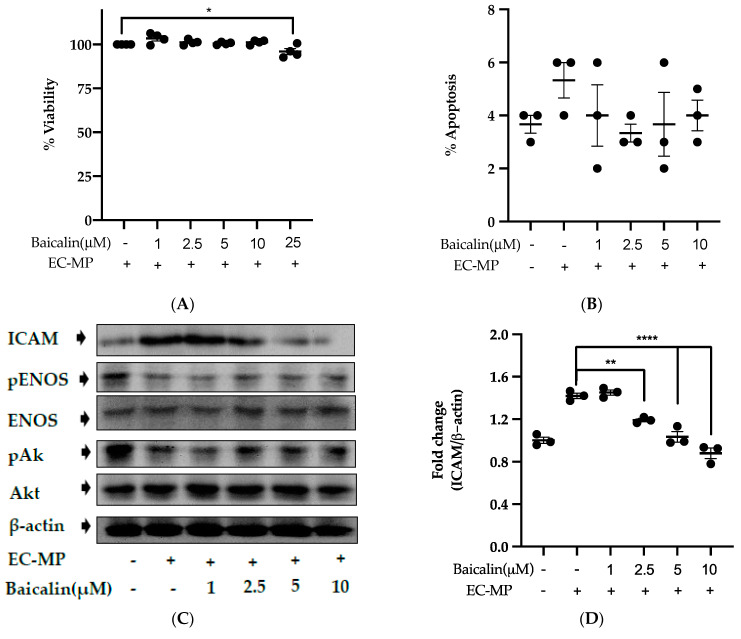

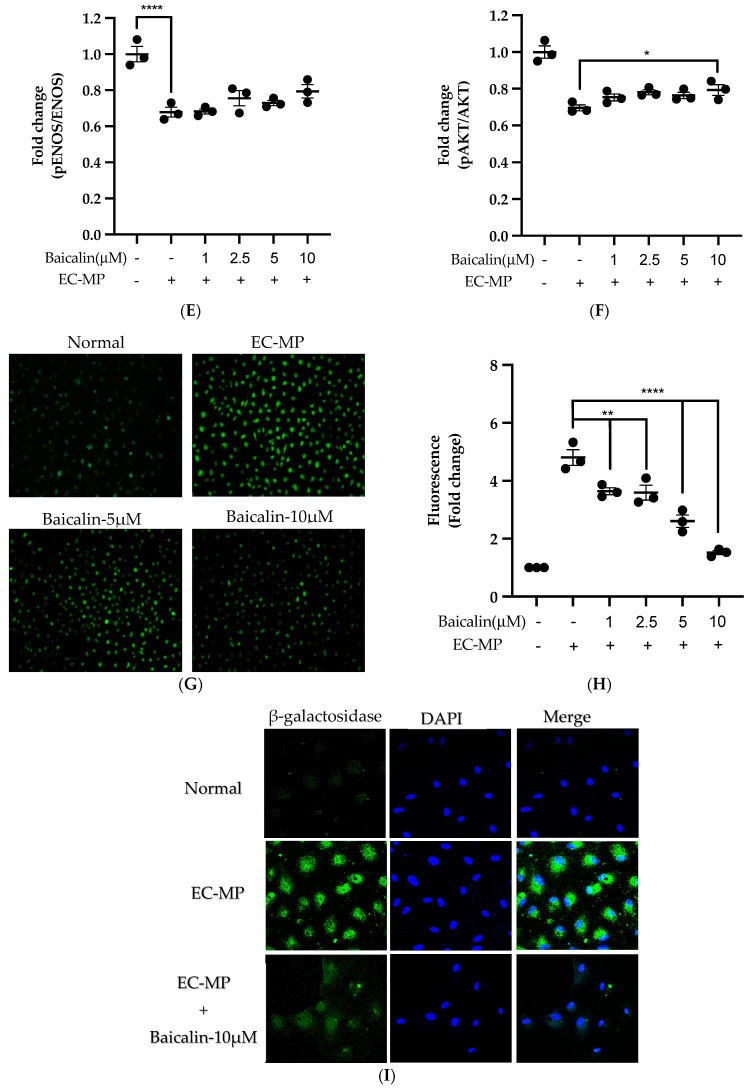
Effect of baicalin on endothelial dysfunction marker. (**A**) EC was incubated with EC-MP and various doses of baicalin for 24 h, followed by MTT solution for 4 h. The absorbance of purple formazan was taken at 540 nm. (**B**) EC treated with or without EC-MP, and a various dose of baicalin was incubated with Annexin V-FITC followed by propodium iodide. Analysis of EC apoptosis was carried out with Arthur^TM^ image-based cell analyzer. (**C**) The cell lysate of EC treated with or without EC-MP and various doses of baicalin for 24 h was subjected to SDS-PAGE for the detection of ICAM, pENOS, and pAkt protein expression by Western blot. β-actin was used for normalization. (**D**) Quantitative data of ICAM fold change. (**E**) Quantitative data of pENOS fold change. (**F**) Quantitative data of pAkt fold change. (**G**) EC was treated with or without EC-MP and a various dose of baicalin for 24 h. DCF-DA was treated to detect the ROS produce by EC indicated by green fluorescence. Images were taken at 20× magnification. (**H**) ROS fluorescence intensity was measured at excitation 485 nm and emission 527 nm. (**I**) EC treated with EC-MP and/or baicalin were labeled with the β-galactosidase (green) and incubated with DAPI for nuclear stain (blue). Fluorescence images were taken at 20× magnification with a confocal microscope. Each value is the mean ± SE of three to four independent experiments, and each independent experiment contains five replicates. *p < 0.05, **p < 0.01, ****p < 0.0001 vs. control (EC-MP alone). The analysis was performed using one-way ANOVA followed by Dunnett multiple comparison test.

Firstly, we evaluated the EC cell viability by the treatment of various doses of baicalin and EC-MP. As shown in Figure 4A, baicalin, at a dose up to 10 µM, did not alter the EC viability compared to normal (baicalin untreated group). Similarly, Figure 4B shows the results of EC-MP induced EC apoptosis. Although EC-MP slightly increases the apoptosis of EC, baicalin slightly decreases the apoptosis of EC, however, there was no statistically significant difference. Endothelial dysfunction results in altered protein expression by EC. As shown in Figure 4C, the protein expression of ICAM was increased, whereas pENOS and pAKT were decreased by EC-MP treatment. In contrast, baicalin treatment results in a significant decrease in protein expression of ICAM and a slight increase in pAKT but no significant difference in pENOS expression (Figure 4D–F). Similarly, the production of ROS in response to EC-MP was increased in EC, while baicalin dose-dependently inhibited the ROS generation (Figure 4G,H). Likewise, EC-MP induced the senescence of EC as revealed by an increase in senescence induced β-galactosidase positive fluorescence staining while baicalin at a dose of 10 μM significantly decrease the β-galactosidase staining (Figure 4I)

### 3.5. Baicalin Inhibits Proliferation of VSMC Induced by VSMC-MP

**Figure 5:**
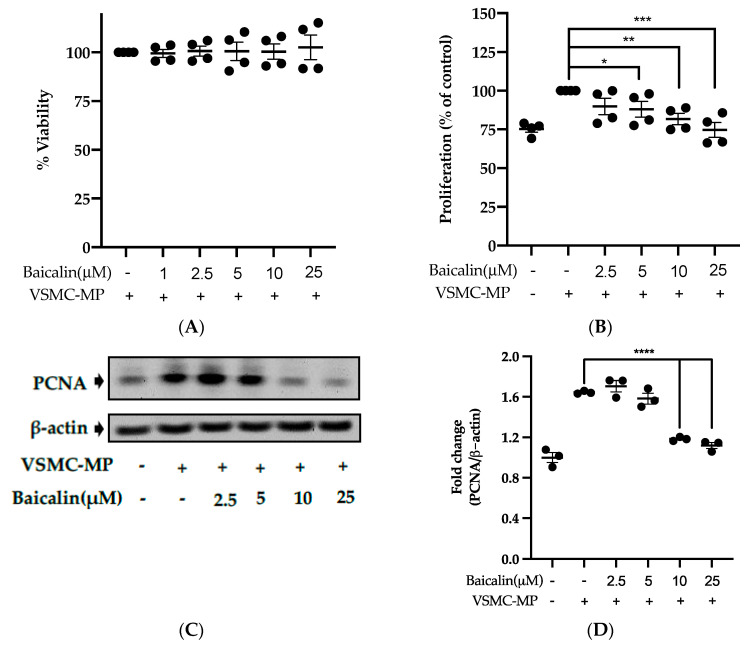

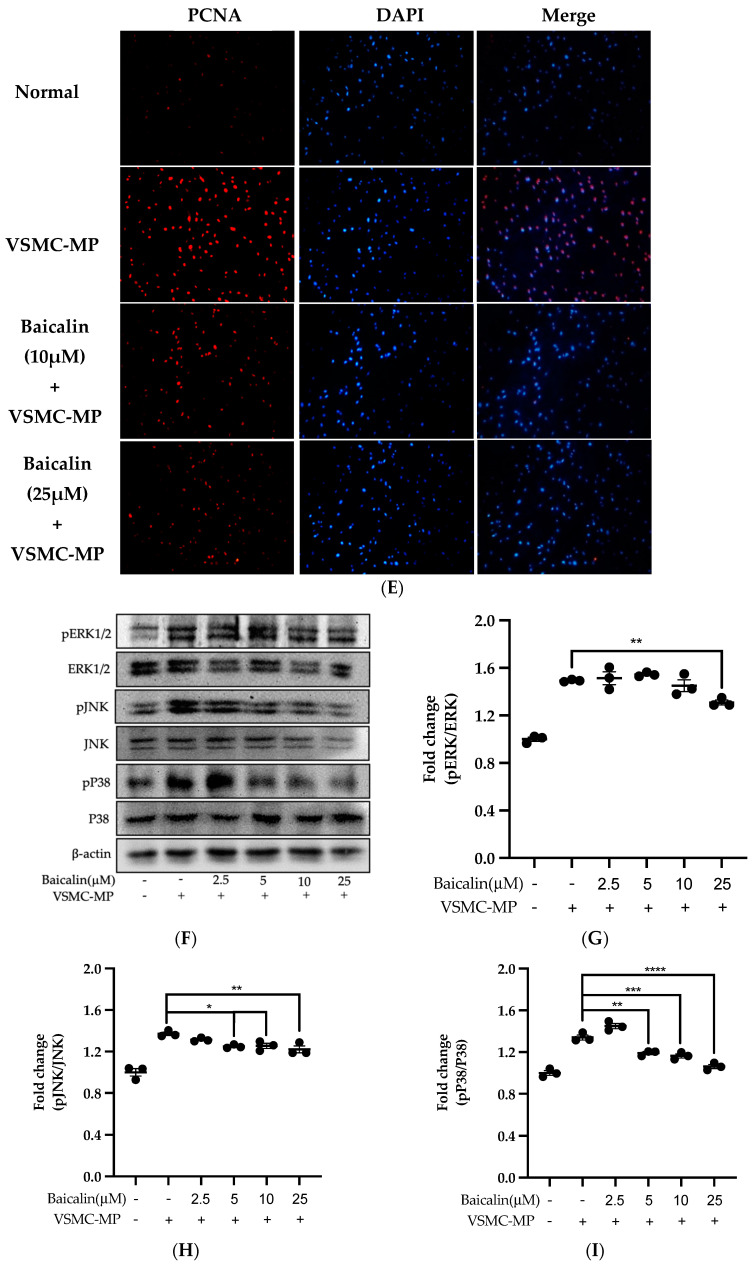
Effect of baicalin on VSMC-MP induced proliferation of VSMC. (**A**) VSMC were incubated with VSMC-MP and a various dose of baicalin for 48 h, followed by MTT solution for 4 h. The absorbance of purple formazan was taken at 540nm. (**B**) VSMC incubated with or without VSMC-MP and various doses of baicalin for 48 h were evaluated for proliferation by MTT method. (**C**) The cell lysate of VSMC treated with or without VSMC-MP and various doses of baicalin for 24 h and was subjected to SDS-PAGE for the detection of proliferative cell nuclear antigen (PCNA) protein expression by Western blot. β-actin was used for normalization. (**D**) Quantitative data of PCNA fold change. (**E**) VSMC were incubated with or without VSMC-MP and baicalin for 24 h. VSMC were exposed to PCNA primary antibody followed by Alexa 555 and incubated with DAPI for nuclear stain. Fluorescence images were taken at 10X magnification with a fluorescence microscope. (**F**) VSMC were incubated with or without VSMC-MP and baicalin for 1 h. The cell lysate was subjected to SDS-PAGE for the detection of a phosphorylated and non-phosphorylated form of ERK1/2, p38, JNK protein expression by Western blot. β-actin was used for normalization. (**G**) Quantitative data of pERK1/2-fold change. (**H**) Quantitative data of pJNK fold change. (**I**) Quantitative data of pP38 fold change. Each value is the mean ± SE of three to four independent experiments, and each independent experiment contains five replicates. *p < 0.05, **p < 0.01, ***p < 0.001, ****p < 0.0001 vs. control (VSMC-MP alone). The analysis was performed using one-way ANOVA followed by Dunnett multiple comparison test.

**Figure 6:**
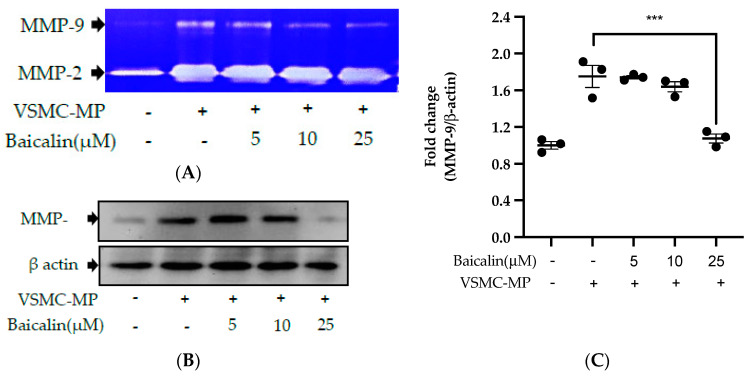

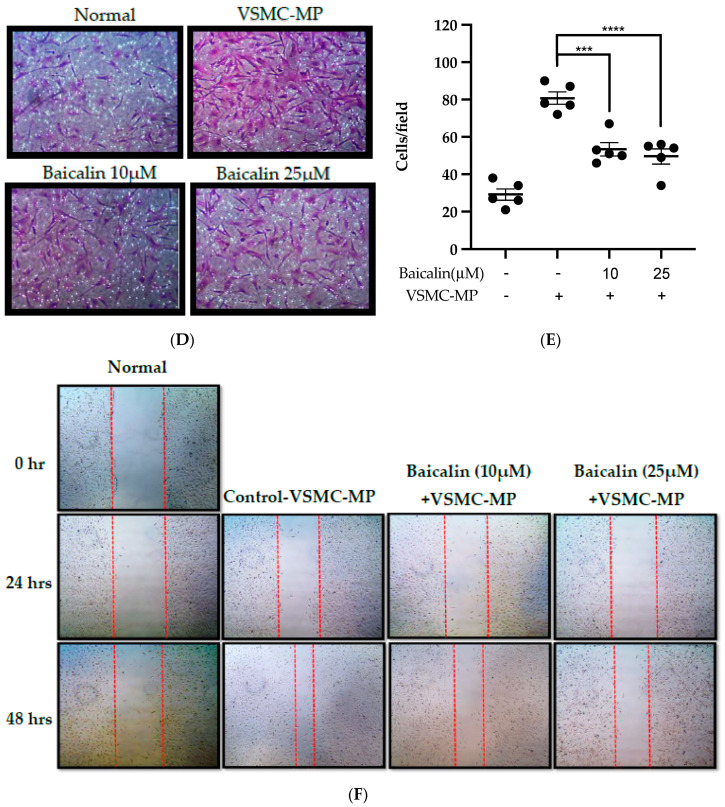
Effect of baicalin on VSMC-MP induced VSMC migration. VSMC were incubated with or without VSMC-MP and baicalin for 24 h. (**A**) Cell supernatant media was subjected to gelatin zymography for the detection of MMP enzymatic activity. (**B**) Cell lysate was subject to Western blot for protein expression of MMP-9. β-actin was used for normalization. (**C**) Quantitative data of MMP-9-fold change. (**D**) VSMCs were induced to migrate through the gelatin membrane barrier in the presence of baicalin and VSMC-MP for 48 h. Images of migrated VSMC were taken after H/E staining (20× magnification). (**E**) The cells on the outer layer after migration were counted in five random positions under the high-power field (HPF). (**F**) The monolayer of confluent VSMC was scratched to create a migrating zone. Photographs were taken at time 0. Then, baicalin was treated at the concentrations of 10 and 25 µM for up to 48 h in the presence of VSMC-MP. Photographs were taken at 24 h and 48 h (20× magnification). Each value is the mean ± SE of three to five independent experiments, and each independent experiment contains five replicates. ***p < 0.001, ****p < 0.0001 vs. control (VSMC-MP alone). The analysis was performed by one-way ANOVA followed by Dunnett multiple comparison test.

Initially, we evaluated the VSMC cell viability by treatment of various doses of baicalin along with VSMC-MP. As shown in Figure 5A, baicalin, at a dose up to 25 µM did not alter the VSMC viability as compared to normal (baicalin untreated group). VSMC-MP cause approximately 25% more proliferation of VSMC than the normal group (VSMC-MP untreated). However, baicalin inhibited the proliferation of VSMC in a dose-dependent manner (Figure 5b). Consistently, the increase in PCNA expression (a marker of VSMC proliferation) by VSMC-MP was inhibited by baicalin in both Western blot (Figure 5C,D) and immunofluorescence experiments (Figure 5E). The overexpression of MAPK signaling pathway proteins is responsible for VSMC proliferation [24]. As shown in Figure 5F, the increase in MAPK protein expression-induced VSMC-MP was inhibited with baicalin treatment. There was a dose-dependent inhibition of pERK 1/2 (Figure 6G), pJNK (Figure 6H), and pP38 (Figure 6I) by baicalin.

### 3.6. Baicalin Inhibits VSMC Migration Induced by EC-MP and VSMC-MP

VSMC-MP increases both enzymatic activity and protein expression of MMP-9, respectively, in VSMC. However, the treatment of baicalin showed dose-dependent inhibition in both gelatin zymography (Figure 6A) and Western blot analysis (Figure 6B,C). In the Boyden chamber assay, VSMC-MP stimulated the migration of VSMC from the upper chamber to the lower chamber. However, baicalin was successful in halting the VSMC migration (Figure 6D,E). Consistently, in another model of cell migration (wound healing assay), baicalin significantly reduced the migration of VSMC to the artificially created wound (migration area) in a dose-dependent manner (Figure 6F).

### 3.7. Baicalin Inhibits ROS Production and Apoptosis in VSMC-MP Induced VSMC

**Figure 7:**
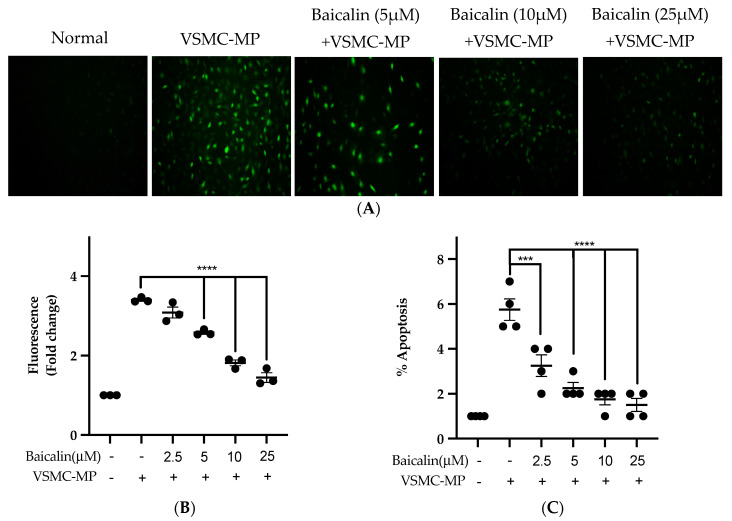
Effect of baicalin on ROS production and apoptosis of VSMC induced by VSMC-MP. (**A**) VSMC was treated with VSMC-MP and various doses of baicalin for 24 h. DCF-DA was treated to detect the ROS produce by VSMC indicated by green fluorescence. Image was taken at 20X magnification. (**B**) ROS fluorescence intensity was measured at excitation 485 nm and emission 527 nm. (**C**) VSMC treated with or without VSMC-MP and various doses of baicalin was incubated with Annexin V-FITC followed by propodium iodide. Analysis of VSMC apoptosis was completed with Arthur^TM^ image-based cell analyzer. Each value is the mean ± SE of three to four independent experiments, and each independent experiment contains five replicates. ***p < 0.001, ****p < 0.0001 vs. control (VSMC-MP alone). The analysis was performed using one-way ANOVA followed by Dunnett multiple comparison test.

Figure 7A shows the activity of baicalin to inhibit ROS production in VSMC-MP-induced VSMC. There was a 4.5-fold increase in ROS production after SMC-MP treatment as compared to the normal group, whereas baicalin dose-dependently reduced the cellular ROS level (Figure 7B). Similarly, the apoptosis of VSMC was increased 5.5-fold, while baicalin treatment decreases the VSMC apoptosis rate (Figure 7C).

### 3.8. Antioxidant Activity of Baicalin

**Figure 8:**
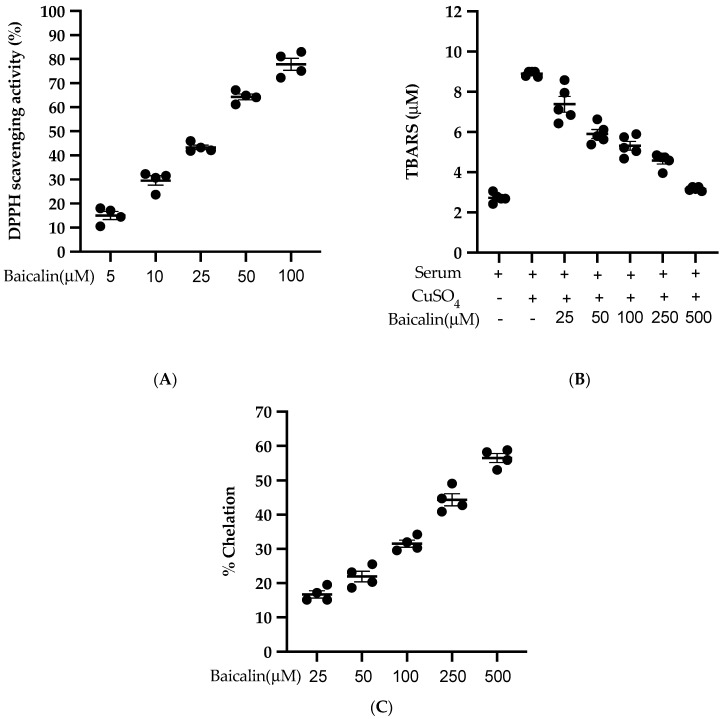
Antioxidant activity of baicalin (**A**) % of DPPH radical scavenging activity was calculated using five different doses of baicalin. An equal volume of baicalin and methanolic solution of DPPH was incubated for 30 min in the dark, and absorbance was taken at 517nm. (**B**) Measurement of thiobarbituric acid reactive substance (TBARS) level was performed by comparison with the standard curve obtained using various dilutions of malondialdehyde (MDA). (**C**) A various dose of baicalin was incubated with FeSO_4_ and 2,2′ bipyridyl. The absorbance of the resulting chromophore was read at 540nm. Each value is the mean ± SE of four-five independent experiments.

Baicalin scavenges the DPPH free radical in a dose-dependent manner with an IC_50_ of 27.21 µM (Figure 8A). Similarly, baicalin also inhibits the lipid peroxidation induced by CuSO_4_ with an IC_50_ of 95.09 µM. (Figure 8B). Likewise, baicalin possesses significant metal-chelating potency with an IC_50_ of 352.04 µM (Figure 8C). This potent antioxidant potency of baicalin could be the possible reason to inhibit the ROS formation in all three cell lines (VSMC, EC, MØ) in vitro.

## 4. Discussion

In our previous study, we provided a lot of evidence of VSMC migration and proliferation and release of inflammatory mediators by activated macrophage leading to the progression of vascular inflammation/atherosclerosis [23,24,36,37,38,39,40,41,42,43]. In those studies, we used an inducer such as TNF-α or PDGF or lysophosphatidic acid to stimulate VSMC migration and proliferation, and LPS for inflammation mediator release from the macrophage. Various cell signaling pathways were targeted in VSMC (pathway of VSMC migration such as MMP-9, cytoskeletal remodeling, and pathway of VSMC proliferation such as MAPKs pathway, PCNA expression) and macrophages (NO production, COX-2, iNOS expression in RAW264.7 cell to prevent the vascular inflammation). In the current study, we use the same inducer (TNF-α -and LPS) to induce microparticle release from VSMC, EC, and MØ to reveal the progression of vascular inflammation by exposing cells with their respective microparticles. Microparticles were successfully isolated from established methods, and they were used for various cell-based assays to reveal their role in vascular inflammation targeting multiple cell signaling pathways.

The MAPK protein family includes ERK1/2, JNK, and p38, which are actively involved in the proliferation and migration of VSMC leading to intimal hyperplasia [41]. In our study, VSMC-MP induced proliferation of VSMC, as shown by the results of the MTT assay and upregulation of MAPK and PCNA protein expression. The treatment of baicalin dose-dependently inhibited the proliferation of VSMC. Similarly, VSMC-MP induced the migration of VSMC in a modified Boyden chamber assay and wound healing assay. VSMC migration was supported by the overexpression of MMP-9 by VSMC-MP. MMP-9 can degrade the extracellular matrix barrier, thereby facilitating the migration of VSMC [36]. In contrast, the treatment of baicalin inhibited the VSMC migration by targeting MMP-9 in a dose-dependent manner. Furthermore, VSMC-MP increased the generation of ROS in VSMC approximately five-fold compared to the untreated group, which is consistent with the same fold of increase in apoptosis of VSMC by VSMC-MP. However, baicalin halted the ROS production and slowed down the apoptosis of VSMC in a dose-dependent manner. One of the reasons to halt the ROS production is because of the potent anti-oxidation activity of baicalin, as shown in our cell-free antioxidant assay (Figure 8A-C). Since microparticles are generated either by cell activation or by cell apoptosis [1], it is possible that by slowing the rate of apoptosis, baicalin can decrease the further microparticle release.

Vascular inflammation is exaggerated by inflammation mediators released by activated macrophages. RAW264.7 is a murine macrophage cell line widely used in the study of atherosclerosis as they play a crucial role in the progression of vascular inflammation through the overproduction of various inflammation mediators [44]. These mediators include excessive NO release via iNOS pathway in response to stimuli such as LPS, overexpression of COX-2 producing inflammatory prostaglandin, NF-κB translocation from cytosol to nucleus, the release of TNF-α and IL-6 [24]. LDL in the blood can easily undergo oxidation by ROS to produce oxidized-LDL, which is engulfed by an activated macrophage and accumulates in a significant amount as a fatty streak, later changing into a foam cell, which is commonly present in atherosclerotic plaque [45,46]. In our study, MØ-MP increases the expression of iNOS and correspondingly increases the production of NO in RAW264.7. Moreover, there was also an increase in COX-2 protein expression, production of ROS, and formation of foam cells in MØ-MP-stimulated RAW264.7. In contrast, the treatment of baicalin remarkably inhibited all these inflammatory mediators.

Endothelial dysfunction is a result of an imbalance between endogenous vasodilator and vasoconstrictor agent. Specifically, the reduction in NO release via the ENOS pathway affects the endothelium-dependent vasorelaxation, a characteristic feature of vascular dysfunction [47]. Damage to the healthy endothelial cells is caused by various noxious agents such as bacterial LPS, ROS, TNF-α, myeloperoxidase and other inflammatory cytokines released by macrophage or neutrophil. All of this agent can trigger EC aging and apoptosis, followed by EC-MP generation. These EC-MP then facilitate the progression of vascular inflammation [20]. EC-MP induce EC aging (senescence), which leads to a decrease in NO production via the ENOS and AKT pathways [31]. We observed that the 24hr treatment of EC-MP to EC leads to senescence of cells (revealed by intense β-galactosidase positive staining). In contrast, the treatment of baicalin significantly decreases the β-galactosidase staining (Figure 1). Furthermore, EC-MP expresses the cell adhesion molecules such as ICAM-1, VCAM, PECAM-1, and VE-cadherin [6,15] possessed from the parent cell which makes them potent inducers of EC dysfunction by facilitating the adhesion of circulating cells such as macrophages to the EC. In the cultured EC, it is reported that the production of NO is controlled by Akt-dependent phosphorylation of eNOS. EC-derived NO helps to maintain vascular homeostasis. Therefore, the inhibition of the Akt pathway or mutation of Akt site on eNOS protein could prevent activation of eNOS leading to altered vascular tone and endothelial function [48].

**Figure 9:**
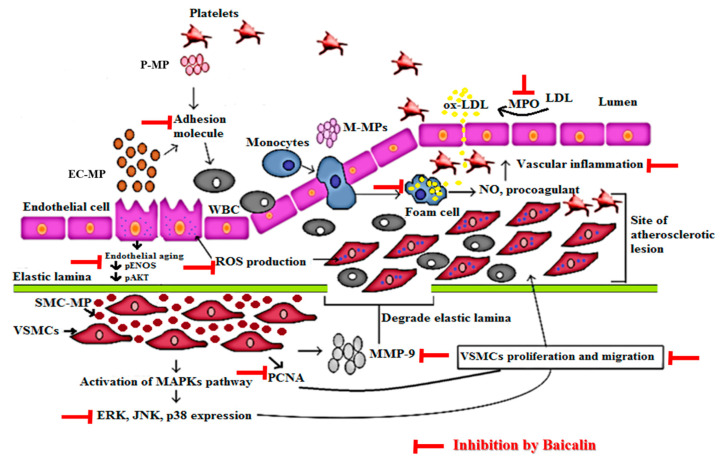
Mechanism of vascular inflammation mediated by microparticles and target of baicalin. Endothelial cell (EC), vascular smooth muscle cell (VSMC), and macrophage cell activation in response to various stimuli such as ROS, LPS, tumor necrosis factor (TNF)-α, interleukin (IL) lead to the production of endothelial microparticle (EC-MP), smooth muscle cell-microparticles (VSMC-MP) and macrophage microparticles (MØ-MP) respectively. EC-MP increases the ROS production in EC, thereby reduce phosphorylated ENOS and Akt expression followed by a decrease in nitric oxide (NO) production (a potent vasorelaxant). VSMC-MP stimulates VSMC to express matrix metalloproteinase-9 (MMP-9), leading to the degradation of the elastic lamina barrier and ease VSMC migration to the site of the atherosclerotic lesion. Furthermore, VSMC-MP also upregulates the mitogen-activated protein kinase pathway (MAPK) (ERK, JNK, P38) pathway and PCNA protein expression leading to proliferation and migration of VSMC to develop vascular inflammation. EC-MP and platelet microparticles (PMPs) possesses cell adhesion molecules that support the adhesion of various circulating cell the endothelial cell followed by altered intimal permeability and vascular lesion at a later stage. MØ-MP activated macrophage to uptake oxidized LDL and change in foam cell. This activated form of macrophage is responsible for the overexpression of nitric oxide (NO) and the procoagulant factor causing vascular inflammation. As a whole, all of this pathway leads to atherosclerosis. The target of baicalin to inhibit vascular inflammation is shown by the red line.

The detailed mechanism of vascular inflammation induced by various microparticles is shown in Figure 9. In our study, EC-MP induced most of the markers of EC dysfunction when treated with EC. The overexpression of ICAM and reduced expression of pAkt by EC-MP was reversed by baicalin treatment. Furthermore, the production of ROS and senescence of EC was also inhibited by baicalin. Collectively, our results support the fact that bacalin lowers the vascular inflammation by targeting abnormalities in three key cell types (EC, VSMC, and MØ) involved in the pathogenesis of vascular inflammation/atherosclerosis. The concept of microparticles inducing disease progression is considered new. Recently, researchers have been studying the various herbal interventions and synthetic compounds to explore the activity of these promising drug candidates to target microparticle-mediated pathological conditions.

A recent study has shown that dietary flavanol intervention lowers endothelial microparticles with an improvement in endothelial function in coronary artery patients [49]. However, this study could not reveal the specific mechanism followed by flavonol to improve endothelial function. Kam et al. showed that curcumin reduced microparticle release from endothelial cells. However, there was no clear indication that endothelial microparticles can act like TNF-α to cause the overexpression of cell adhesion molecules, which is an important parameter during vascular inflammation [50]. Another study by Schwarz et al. stated that red wine prevents the acute negative vascular effect of smoking. This clinical study concludes that smoking leads to elevation of endothelial-, platelet-, monocyte-, and leukocyte-derived microparticles while consumption of red wine lowers the microparticle levels. However, this study’s limitation was a lack of evidence to prove the exact molecular mechanism of how the levels of microparticles were lowered by the beneficial activity of red wine [51]. Nevertheless, these herbal candidates can be a potential option for preventing disease caused by the overproduction of microparticles. Our study showed the molecular mechanism followed by baicalin to target various pathways in cell types (EC, VSMC and MØ) involved in vascular inflammation. Taken together, our finding suggests baicalin as a promising compound with the potential to ameliorate the features of vascular inflammatory disease. However, our study has few limitations, and it opens the platform for further studies and guidance for researchers working with microparticles. First, all our evidence is experimental data from in vitro studies from various cell lines. It would be more interesting to study and validate the in vitro finding with the clinical setting of vascular inflammation/atherosclerosis by carrying out experiments in primary cells isolated from the healthy controls and patients with acute coronary studies. Second, the levels of microparticles are high in the diseased state but not the same in all individuals. Therefore, it is difficult to find the exact clinically relevant dose of microparticles that can induce the features of vascular inflammation in in vitro experiments. It is essential to carry out the dose–titration study to investigate the clinical relevance dose of microparticles in various disease states before carrying out the in vitro and animal model studies.

## 5. Conclusions

Baicalin possesses potent antioxidant activity, inhibiting VSMC-MP-induced VSMC proliferation via the PCNA and MAPK signaling pathways, and migration via the inhibition of MMP-9. Baicalin also attenuated the MØ-MP-induced vascular inflammation by the downregulation of potent inflammatory mediators; NO, ROS, iNOS, COX-2, and foam cell formation in RAW264.7. Baicalin inhibits endothelial dysfunction by downregulating ROS production and ICAM expression while upregulating pENOS and pAkt in endothelial cells. These signify that baicalin can be a potential therapeutic option for the prevention of vascular inflammatory disorders such as atherosclerosis.
